# Hesperidin Promotes Osteogenesis and Modulates Collagen Matrix Organization and Mineralization In Vitro and In Vivo

**DOI:** 10.3390/ijms22063223

**Published:** 2021-03-22

**Authors:** Patricia A. Miguez, Stephen A. Tuin, Adam G. Robinson, Joyce Belcher, Prapaporn Jongwattanapisan, Kimberly Perley, Vinicius de Paiva Gonҫalves, Arash Hanifi, Nancy Pleshko, Elisabeth R. Barton

**Affiliations:** 1 Division of Comprehensive Oral Health, School of Dentistry, University of North Carolina at Chapel Hill, Chapel Hill, NC 27599, USA; viniciusodonto2007@hotmail.com; 2 Oral and Craniofacial Health Sciences, University of North Carolina at Chapel Hill, Chapel Hill, NC 27599, USA; satuin@gmail.com (S.A.T.); agrobins@live.unc.edu (A.G.R.); cocabprapaporn@gmail.com (P.J.); 3 Cabrini University, Philadelphia, PA 19019, USA; jybelcher@gmail.com; 4 Department of Periodontics, School of Dental Medicine, University of Pennsylvania, Philadelphia, PA 19104, USA; kimberlyperley@gmail.com; 5 Department of Bioengineering, Temple University, Philadelphia, PA 19122, USA; ahanifi@gmail.com (A.H.); npleshko@temple.edu (N.P.); 6 Department of Applied Physiology and Kinesiology, University of Florida, Gainesville, FL 32611, USA; erbarton@ufl.edu

**Keywords:** collagen, bone, osteogenesis, hesperidin, extracellular matrix, regeneration, critical-sized defect, bone morphogenetic protein

## Abstract

This study evaluated the direct effect of a phytochemical, hesperidin, on pre-osteoblast cell function as well as osteogenesis and collagen matrix quality, as there is little known about hesperidin’s influence in mineralized tissue formation and regeneration. Hesperidin was added to a culture of MC3T3-E1 cells at various concentrations. Cell proliferation, viability, osteogenic gene expression and deposited collagen matrix analyses were performed. Treatment with hesperidin showed significant upregulation of osteogenic markers, particularly with lower doses. Mature and compact collagen fibrils in hesperidin-treated cultures were observed by picrosirius red staining (PSR), although a thinner matrix layer was present for the higher dose of hesperidin compared to osteogenic media alone. Fourier-transform infrared spectroscopy indicated a better mineral-to-matrix ratio and matrix distribution in cultures exposed to hesperidin and confirmed less collagen deposited with the 100-µM dose of hesperidin. In vivo, hesperidin combined with a suboptimal dose of bone morphogenetic protein 2 (BMP2) (dose unable to promote healing of a rat mandible critical-sized bone defect) in a collagenous scaffold promoted a well-controlled (not ectopic) pattern of bone formation as compared to a large dose of BMP2 (previously defined as optimal in healing the critical-sized defect, although of ectopic nature). PSR staining of newly formed bone demonstrated that hesperidin can promote maturation of bone organic matrix. Our findings show, for the first time, that hesperidin has a modulatory role in mineralized tissue formation via not only osteoblast cell differentiation but also matrix organization and matrix-to-mineral ratio and could be a potential adjunct in regenerative bone therapies.

## 1. Introduction

Grafts for bone regeneration and repair are frequently used but have limitations and variable degrees of success. The use of bone autografts typically involves some level of morbidity from the donor site, while allografts and xenografts can present limited acceptance from patients and variable osteoinduction levels. Alloplasts, such as ceramic or metal, sometimes lead to a low level of bone integration and fibrous encapsulation [1,2]. A more modern approach of “tissue engineering” has been developed to improve the success rates of bone regeneration. This strategy consists in combining a biocompatible scaffold (xeno- or alloplastic in nature) along with cells and/or, more frequently, with osteogenic growth factors such as bone morphogenetic proteins (BMPs) or platelet-derived growth factor (PDGF) [3,4]. At present, the clinical use of such combined therapies still faces serious limitations including, but not limited to, the requirement of high quantities of growth factor, difficulties in retaining the molecules in situ, excessive inflammation, occasional ectopic ossification and high cost [5,6,7]. Thus, development of a more efficient and cost-effective means for bone regeneration and repair is still of critical importance.

An emerging strategy for enhancing overall health has been to increase intake of naturally derived compounds. Data from the 2012 National Health Interview Survey (NHIS) found that non-vitamin and non-mineral natural products are the most popular complementary health methods used by Americans [8], and this approach has been on the rise for about a decade. Several botanicals/phytochemicals, which are present in plants and foods, have been historically used for a wide range of health problems, are available over the counter and are of low cost. A large portion of the data available for phytochemicals refers to them as antioxidants as they may modulate mechanistically various pathologies associated with oxidative stress such as cancer, cardiovascular disease and chronic inflammation [9,10,11]. Today, the most studied phytochemicals are flavonoids. Phenolic compounds or flavonoids are sub-classified as anthocyanidins, flavonols, flavanones, flavanols, flavones and isoflavones [12]. Despite significant data on flavonoids, their effects on cell differentiation and function as well as collagen matrix characteristics specifically related to osteogenesis have not been widely investigated. Previously, we and others showed that the flavonoid proanthocyanidin (PA) is an effective type I collagen cross-linker that prevents demineralization of dentin with some limited anti-bacterial activity [13,14]. Unfortunately, some types of cells do not respond well to PA, resulting in halting of proliferation and viability, which limits its utilization [15]. Flavanones present in citrus fruits such as hesperidin (H; 3′,5,7-trihydroxy-4′-methoxy-flavanone-7-rhamnglucoside), in particular, have been shown to stimulate osteoblast differentiation through activation of BMP signaling via its metabolite hesperetin [16]. Pre-clinical studies have demonstrated that H intake results in bone density protection in senescent and ovariectomized rats as well as reduction in oxidative stress and overall lipid content [17,18,19,20]. Recently, our group investigated the use of grape seed extract and grapefruit extract (the latter rich in H) in bone healing of extraction sockets and found that grapefruit extract use led to downregulation of inflammatory genes such as interleukin (IL)-1 β, IL-6 and CXCL2 [21]. Here, we have verified the non-deleterious effects of H in cell proliferation and viability and have investigated the effects of this promising phenolic compound on pre-osteoblastic cell differentiation, on the quantity and quality of mineralization, on the quality of the organic type I collagen rich matrix in vitro, which is critical for bone properties, as well as its potential bone-inducing role and bone matrix quality in vivo.

## 2. Results and Discussion

In this study, we explored the potential direct effects of H on pre-osteoblastic cell proliferation (MC3T3-E1, MC) and viability, cell differentiation, quality of collagen matrix produced and mineralization in an osteoblastic cell culture system. Furthermore, we evaluated a potential osteogenic and matrix quality influence of H when locally delivered to a critical-sized defect rat mandible model.

**Figure 1:**
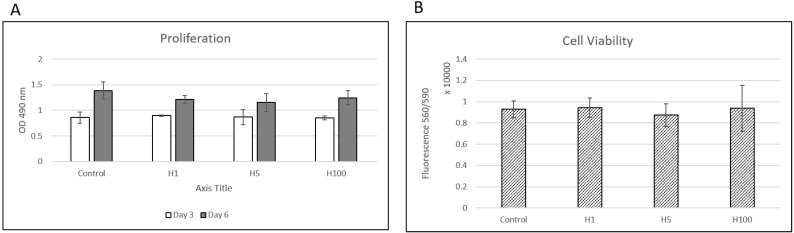
**MC3T3-E1** cell proliferation (**A**) and viability (**B**) upon treatment with hesperidin (H) at concentrations of 1, 5 (H5) and 100 (H100) μM show no significant differences (*n* = 3/group, *p* > 0.05).

At all timepoints examined, there were no significant differences in cell proliferation between the control and treated groups at days 3 and 6. The results were also confirmed by manually counting the cells at days 2 and 5. For cell viability, H was found not to affect cell viability (i.e., no cell death detected) at any of the concentrations tested (Figure 1).

**Figure 2:**
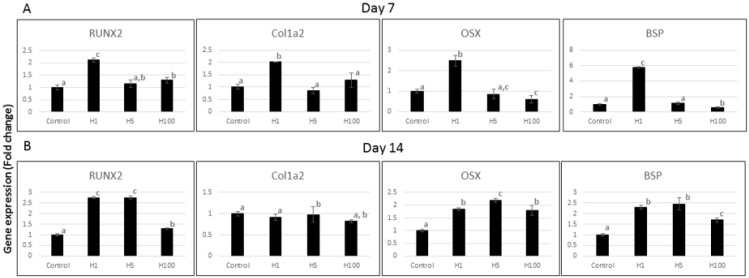
Effect of hesperidin (H) on gene expression of osteogenic markers of MC3T3-E1 pre-osteoblast cells at 7 (**A**) and 14 (**B**) days as analyzed by qRT-PCR. Different superscript letters (a, b, c) indicate statistical difference among groups (*p* < 0.05). H, on day 7, at a dose of 1 µM (H1) showed consistent elevation of osteogenic markers compared to control. On day 14, both lower concentrations of H (1 and 5 µM) showed significantly more elevated *Runx2, Osx and Bsp* (*n* = 3/group).

Changes in gene expression for key markers of osteogenic differentiation were evaluated in MC cells exposed to H for 7 or 14 days by quantitative real time (qRT)-PCR data. As shown in Figure 2, there were time- and dose-dependent effects on gene expression. *Runx2* expression significantly increased at days 7 and 14 with H exposure, particularly at lower doses (1 and 5 µM). *Col1a2* expression increased about two-fold at day 7 with low H (1 µM), and showed no response at day 14. *Osx* was increased significantly with H at the 1 µM (H1) dosage at day 7 and for all H concentrations at day 14. *Bsp*, also a late marker of osteogenesis as *Osx* and indicative of mineralization, was close to six-fold higher with H1 at day 7. At day 14, all H concentrations showed statistically significant increases in *Bsp* expression. The results indicate that H promotes key osteogenesis markers such as *Runx2*, *Osx* and *Bsp* in proliferation and early mineralization phases of osteoblast culture, with some being more positively affected by the lower dose of H. The sustained increase in *Runx2* and *Osx,* which are essential transcriptional factors in bone formation, may indicate more stable promotion of osteogenesis when H is present [22,23]. A recent study demonstrated the promising role of an H metabolite in promoting bone formation in an in vivo model of tibia fracture, which validates our in vitro osteogenesis data [24].

**Figure 3:**
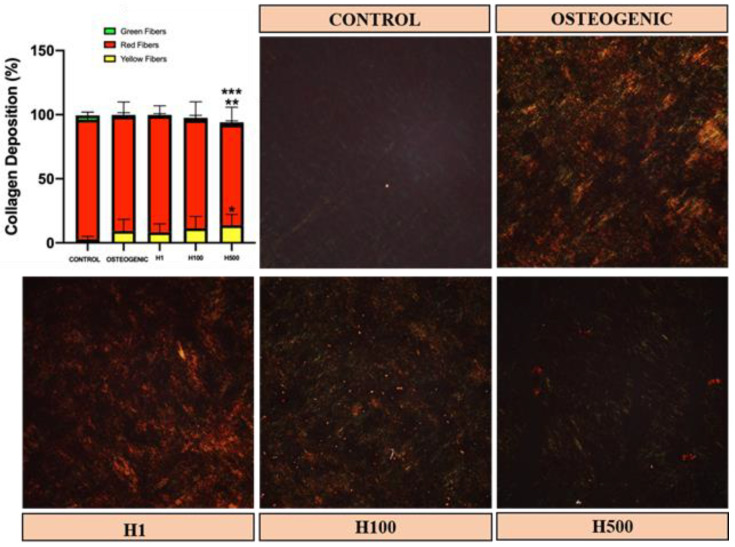
Picrosirius red (PSR) staining of MC3T3-E1 cell-produced collagenous matrices after culture for 21 days with and without hesperidin (H) in the medium (1, 100 or 500 μM). Three independent experiments produced similar results and one representative polarized image of each condition is shown. The collagen matrices in H-treated samples maintained collagen at similar levels except for H500. H500 had reduced collagen accumulation and a significant decrease in red fibrils compared to other groups of treatment (red-color signal indicates presence of mature collagen) (** indicates difference compared to osteogenic medium alone at *p* < 0.05; *** indicates difference compared to osteogenic and other H groups at *p* < 0.05). Yellow fibrils (immature) increased in H500 (* indicates statistical difference compared to control). No green signal was detected in cultures (green depicts the least mature fibril, typical of early collagen deposits). All cells had similar viability as determined by CellTiter-Blue Cell Viability Assay (*n* = 3, 10X magnification).

In vitro, the organization of collagen matrix in the untreated controls was uniform but lightly colored under picrosirius red (PSR) staining and polarized light, indicating a thin and/or loose matrix at day 21 (Figure 3, dark colored background control). Typical staining of collagen via PSR shows red, yellow and green colors, which have been associated with collagen packing and post-translational modifications [21,25]. Red indicates more mature/well-packed/organized collagen, and green indicates least mature/loose-packed/disorganized collagen [26]. The collagen matrices in in vitro H-treated samples were organized and well packed as was the case for osteogenic media alone. However, the increase in H dose to 500 µM decreased the total amount of collagen deposited and increased the amount of yellow-colored fibrils. These results demonstrate that H can modulate the organization and maturation of collagen matrix secreted by osteoblasts, although the mechanism of such an influence is still unknown. Previously, it has been shown that H could add mechanical strength to skeletal bone via dietary route in rodents, which can be a result of collagen maturation effect [18].

**Figure 4:**
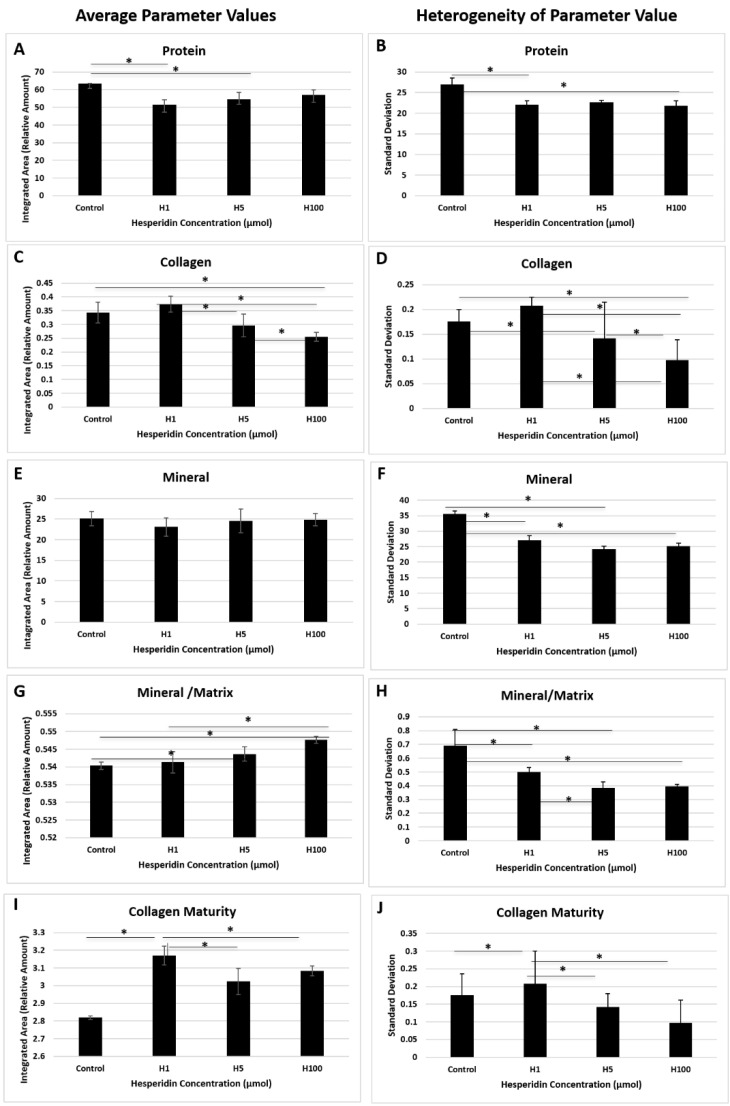
Average FT-IR-derived parameters for control and treated samples demonstrate matrix composition and quality changes. Total protein was affected by hesperidin (H)1 (1 µM) and H5 (5 µM) doses (**A**) and collagen matrix amount was reduced significantly with H5 and H100 (100 µM) doses (**C**) (* represents statistical difference *p* < 0.05). Standard deviation or heterogeneity plots indicate the variation in matrix composition and quality within the sample. Parameters from H-treated samples were less heterogeneous (**B**,**D**,**F**,**H**–**J**), i.e., a more homogenous distribution of tissue components. Mineral-to-matrix ratio was significantly increased with higher doses of H (**G**).There were no changes in average mineral content among samples (**E**) (*n* = 3/group).

H treatment of MC cultures for 21 days resulted in significant matrix and mineral differences among the treated and control groups for Fourier-transform infrared spectroscopy (FT-IR)-determined total protein (Figure 4A) and collagen (Figure 4C,I). Collagen content peaked at 1 µM (Figure 4C,D) and then decreased with increasing H concentration. This agrees with qRT-PCR data showing collagen expression being the highest for H at 1 µM (H1). Collagen maturity was significantly lower for the control samples compared to the H-treated samples (Figure 4I,J), particularly for H1. There was no difference in the average mineral content among all samples (Figure 4E). However, mineral content normalized to the total protein of the tissue (min/mat) showed significant increases with increasing H concentration of cultured tissues (Figure 4G). The mineral crystallinity did not change significantly among treated and control samples (control ~1.3; H1 ~1.1; H5 ~1.1 and H100 ~1.1 in integrated area/relative amounts). Parameters from treated samples were less heterogeneous (Figure 4B,D,F,H,I,J), i.e., there was a more homogenous distribution of tissue components for most parameters compared to controls. The mechanism of H on cell activity and matrix production is not well known, but cells responded by producing a more homogenous matrix compared to the control group.

A recent study looked at the effect of H interaction with atelocollagen by NMR. It was suggested that H interacts with collagen without significantly changing the triple-helical structure. However, it may bind at the proteolytic cleavage site, which could confer increased resistance to degradation and, thus, slow remodeling. This could influence homogeneity as well as the maturity of the matrix deposited [27]. Other studies have shown that collagen degradation is inhibited by H treatment [28,29,30]. If this was the case, then collagen would accumulate regardless of changes in expression. As we observed an early increase in *Col1a2* expression, both mechanisms could contribute to the overall increase in collagen levels that we have observed in FT-IR. Further characterization of H-exposed collagen matrix deposition, biochemical modifications and mineralization is warranted. Special attention should be given to effects on collagen post-translational modifications, stability and turnover of collagen, mechanical properties and in vivo response of cells and connective tissues to H.

**Figure 5:**
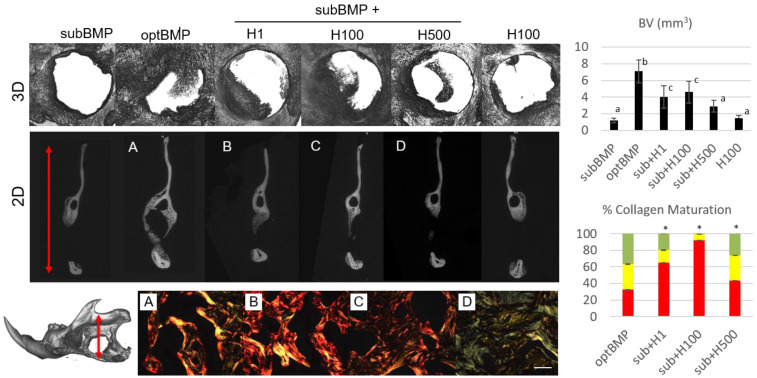
In vivo study of bone regeneration modulation by local delivery of bone morphogenetic protein 2 (BMP2) and hesperidin (H) in a rat mandible critical-sized defect (*n* = 4/group). Microcomputed tomography (µCT) 3D images (top row) show increased bone formation with use of a large dose of BMP2 (optimal dose of 1 µg of BMP2, optBMP) compared to a small dose (suboptimal dose of 100 ng of BMP2, subBMP). Different superscript letters on the top-right chart (BV, bone volume) indicate statistical difference among groups (*p* < 0.05). When subBMP was delivered with three different doses of H, a significantly greater amount of newly formed bone (NFB) was found for subBMP+ H1 (1 µM) and H100 (100 µM) compared to subBMP alone (3D and 2D µCT panels and BV graph). The 2D µCT view is a coronal cross-section of the 5-mm mandible defect (rat mandible depicted on bottom left with red arrow indicating where the 2D cross-section was taken). The 2D view provides additional understanding of the bone fill within the defect as compared to the 3D view, which is a median plane perspective that does not show the ectopic nature of optBMP. The NFB for H-treated samples was found to be within the constraints of the defect (µCT 2D panels **B**–**D**) as opposed to optBMP (**A**). BV for H100 alone was not statistically different from control subBMP (top-right bar graph). Bottom-right chart shows the effect of H on collagen organization of the NFB during BMP-induced bone formation. Polarized light images (20×, bar 200 µm) of PSR-stained NFB (bottom row) highlight differences in the organization/maturation of the bone matrix as there was an increase in more mature collagen (red fibrils) in H-treated samples with lower doses of H (B and C) (* in bar graph indicates statistical difference in red fibril percentage compared to optBMP, *p* < 0.05). SubBMP and H100 PSR alone were not quantified due to the limited amount of NFB. (*n* = 4 images/animal).

Our in vivo study shows that H treatment of a collagen scaffold combined with loading a small dose of BMP2 (first named as a suboptimal, subBMP, a 100 ng dose that is not able to generate bone filling during healing as per Arosarena and Collins) can form well-controlled, defect-confined bone filling up about 20–60% of the defect [31]. A dose of H of 100 µM combined with subBMP showed the highest bone volume (BV) capability (average of ~45%). Figure 5 shows representative microcomputed tomography (µCT) images and BV quantifications of all groups tested. A large dose of BMP2 (called optimal, optBMP, a 1 µg dose that forms rapid bone tissue to heal the defect) shows a larger bone volume than H-treated bone defects, although of ectopic nature (not within the constraints of the 5-mm mandible defect), including invasion of muscle which is not a desirable outcome (Figure 5) [31,32]. Our group has previously confirmed the effects of the collagen scaffold (Nitta Gelatin, Japan) alone and with BMP2 as used in the present study. We previously demonstrated there is high turnover of BMP2 associated with optBMP dose with increased osteoclast numbers present in the newly formed bone (NFB) area as compared to other bone-inducing molecules [33,34]. In this study, PSR staining in vivo indicates that the pattern of collagen maturation varied when a large dose of BMP2 was used vs. a low dose of BMP2 + H. The PSR data demonstrate that there were fewer mature (red) collagen fibrils in optBMP-induced bone vs. subBMP + H. The H1 and H100 doses, when used with subBMP, showed the highest percentages of mature collagen fibrils within the NFB.

BMP2 has been shown to form large bone volumes quickly and cause increased levels of inflammation and even cancer [35]. Ideally, an osteogenic molecule should induce bone within the constraints of the bone defect and induce minimal inflammation and immune reaction. Via its possible osteogenic [24], anti-resorptive [20] and anti-inflammatory effects [36], H may be modulating bone formation in the mandible model presented. Our study suggests that H is a promising agent for local delivery in bone defects and can potentially improve bone organic matrix and, consequently, mechanical properties in the long term compared to BMP2 therapy alone. H alone did not induce significant bone formation as compared to the control (Figure 5); thus, we concluded that addition of a sub-optimal dose of BMP2 is needed in conjunction with H if this phytochemical is to be delivered locally. It is important to note that H is largely non-soluble in aqueous solutions and required DMSO for dissolution in the vehicle used in our study (phosphate-buffered saline, PBS) for all concentrations. In vivo, variations in solubility may lead to inconsistent results. Thus, although the results of our in vivo study are promising, the solubility and delivery of this compound in vivo were not evaluated. The use of H for local delivery warrants the investigation of a controlled release system such as nanoparticles, branched polymers or hydrogels with predictable rates of degradation to facilitate its solubility and to ensure cell and matrix uptake. In an optimized delivery system, a very low dose of BMP could be incorporated as well, which would minimize undesirable BMP-triggered effects.

Under the conditions of this study, the treatment of MC3T3-E1 cells particularly with lower doses of H resulted in overexpression of osteogenic markers, deposition of more organized/well-packed and homogeneous collagen matrix and a favorable mineral-to-matrix ratio. This suggests that H treatment may be beneficial in terms of promoting mineralization and bone quality in vivo. When we analyzed its potential to form new bone in vivo, we observed that after 2 weeks, there was evidence of greater induction of bone formation within the mandible defect as compared to a low dose of BMP2 alone, and not of ectopic nature as in the case of a higher dose of BMP2. A naturally sourced promoter of osteogenesis, mineralization and bone matrix quality may not only be a cost-effective therapy in bone regeneration but also a well-accepted type of intervention by the public seeking care in orthopedics, general medicine and dentistry. This study warrants further investigation on the mechanistic influence of H in BMP function, bone formation, optimization of H delivery in situ as well as long-term evaluation of bone quality and quantity induced by H vs. other osteogenic molecules currently on the market.

## 3. Materials and Methods

### 3.1. Cell Culture, Proliferation and Viability

MC pre-osteoblastic cells (subclone 4) were purchased from American Type Culture Collection (Manassas, VA, USA) and were grown in α-minimum essential medium (α-MEM, Gibco, Carlsbad, CA, USA) containing 10% fetal bovine serum (FBS, Atlanta, Lawrenceville, GA, USA) and supplemented with 100 units/mL penicillin G sodium and 100 µg/mL streptomycin sulfate in a 5% CO_2_ atmosphere at 37 °C. The medium was changed twice a week. This cell line was chosen as the cells undergo differentiation and mineralization upon addition of β-glycerophosphate and ascorbic acid while presenting with natural expression of BMPs. Addition of recombinant BMP to MC cell cultures was not performed due to inherent BMP expression as well as the demonstrated effect of exogenous BMP addition on cell proliferation, collagen cross-linking pattern and maturation, which would affect our analyses [37,38].

In order to assess the cell proliferation of MC cells in response to the natural compound H (Acros Organics, Belgium), we utilized the CellTiter 96 AQ_ueous_ Non-Radioactive Cell Proliferation Assay (Promega, Madison, WI). Cells were plated at a density of 1 × 10^4^ cells/well in 96-well tissue culture plates. The plates were incubated at 37 °C in the presence of 5% CO_2_. Forty-eight hours after plating, media were replaced and supplemented with no treatment or 1, 5 or 100 μM of H in 0.5% DMSO (*v/v*). CellTiter Reagent was added to each well and the plates were incubated, as per the manufacturer’s instructions up to 6 days. After incubation, absorbance values representing the amounts of formazan compound produced by metabolically active cells at 490 nm were recorded using a Tecan Infinite PRO 200 NanoQuant (Tecan Group Ltd., Switzerland). For cell viability, the CellTiter-Blue Cell Viability Assay (Promega) was used and fluorescence values at 560_Ex_/590_Em_ were recorded using a Molecular Devices SpectraMax M5 Plate reader (Molecular Devices, Sunnyvale, CA, USA). These experiments were repeated in triplicate.

### 3.2. Quantitative Real-Time Polymerase Chain Reaction (qRT-PCR)

Cells were plated onto 35-mm dishes at a density of 2 × 10^5^ cells/dish. After reaching confluence (day 0), the medium was replaced with osteogenic media (cell medium above with supplementation of 50 µg/mL ascorbic acid and 2 mM β-glycerophosphate) with and without 1, 5 and 100 µM H. At days 7 and 14 of culture, changes in gene expression were calculated [39]. These time points have been consistently used by our group based on the differentiation and mineralization pattern of MC cells in our laboratory with the proliferation/differentiation stage in the first week, the early mineralization stage in the second week and the later mineralization stage after 21 days [38,40].

Briefly, at the end of 7 (proliferation stage) and 14 days (early mineralization stage), total RNA was extracted using TRIzol reagent (Invitrogen, Carlsbad, CA, USA) according to the manufacturer’s protocol. Briefly, 2 µg of RNA was used to synthesize the first-strand cDNA using an Ominiscript RT kit (Qiagen, Valencia, CA, USA). qRT-PCR using TaqMan Gene Expression Assays (Applied Biosystems, Foster City, CA, USA) was performed according to the manufacturer’s instructions using an AB Step One Plus Real-time PCR System (Applied Biosystems). Sequence-specific PCR primers were utilized for the following osteogenic factors: *Cbfa1/Runx2* (Applied Biosystems, Mm00501584_m1), *Col1a2* (Mm00483888_m1), *Osx* (Mm00504574_m1) and *Bsp* (Mm00492555_m1). Changes in gene expression were calculated using relative quantification of a target gene normalized to the endogenous GAPDH (Mm99999915_g1) control (2-ΔΔCT method). This experiment was performed in triplicate and results were confirmed by three independent experiments [41].

### 3.3. Collagen Organization/Maturation In Vitro by Picrosirius Red (PSR) Staining

In order to assess the collagen organization of MC cell-secreted matrices in response to H, collagen histochemistry was examined by PSR staining. Cells were plated at a density of 3 × 10^3^ cells/well in Lab-Tek II Chamber Slides (Nunc, Rochester, NY, USA) in α-MEM (Invitrogen) containing 10% FBS (Thermo Fisher Scientific, Waltham, MA, USA). Chambers were incubated at 37 °C in the presence of 5% CO_2_. Twenty-four hours after plating, the medium was replaced with one supplemented with 50 μg/mL ascorbic acid and either no treatment or 1, 100 or 500 μM H, the same concentrations as planned for in vivo studies. Cultures were returned to the incubator and monitored daily with change of medium and treatment every 48 h until 21 days of culture. Prior to staining, cultures were washed twice with warm PBS and fixed with 10% formalin for 30 min. Matrices were incubated in 0.1% (*w/v*) Sirius red in saturated picric acid solution for 30 min at room temperature. This was followed by rinsing with distilled water overnight, dehydration and mounting. The slides were imaged under bright field and polarizing light using a Leica DMR microscope (Buffalo Grove, IL, USA). This experiment was repeated in triplicate. PSR images at 20× magnification were analyzed using a custom generated algorithm in MATLAB ^®^ R2016a (Mathworks, Natick, MA, USA) as we previously reported [21,26]. The percent area of red, yellow and green color signals was normalized to the total color signal for each sample. Cells were confirmed viable with the CellTiter-Blue Cell Viability Assay (Promega) as described above and concomitant to PSR experiments (*n* = 3).

### 3.4. Analysis of Mineral and Protein by Fourier-Transform Infrared Spectroscopy Imaging (FT-IR)

Analysis of MC cell matrices treated with H at concentrations of 1, 5 and 100 µM in osteogenic medium for 21 days was performed using FT-IR [42,43,44]. FT-IR image analysis provides a distribution of the sample composition where each pixel (50-µm size, set as the spatial resolution) contains a unique spectrum. Composition parameters measured using integrated areas and peak height ratios obtained from the spectral image also provide a distribution map of the individual parameters that can be used to generate a histogram of the tissue components. From the histograms, mean values of the parameters and standard deviations (related to histogram width at half height, termed heterogeneity) were assessed. The heterogeneity parameter relates to the uniformity of the distribution of each component. Average values and standard deviation (heterogeneity) were analyzed for significant differences for each parameter.

MC cells were plated at a density of 7.5 × 10^4^ in 35-mm tissue culture-treated dishes in osteogenic medium as described above. Approximately 48 h later, osteogenic media were changed to those containing either no compound or the three concentrations of H and returned to the incubator. Media and treatment were replaced every 3–4 days until 21 days of culture. On day 21, media were discarded and cultures were rinsed with PBS twice. After the final PBS rinse, fixed culture layers were treated with ethanol to reduce their adhesion to the plastic substrate and carefully separated from the plastic substrate for placement onto silicon wafer surfaces for data collection. FT-IR data were collected in transmittance in the mid-IR spectral region, 4000–750 cm^−1^, at 8 cm^−1^ spectral resolution and 50 µm spatial resolution using a Spectrum Spotlight 400 FT-IR imaging spectrometer (Perkin Elmer, Shelton, CT, USA).

Infrared spectra were analyzed using ISys 5.0 software (Malvern Instrument, Columbia, MD, USA) to determine tissue composition and mineral content and quality. Total protein, collagen and mineral contents were assessed by the integrated areas under the amide I (1718–1594 cm^−1^), the 1338 (1356–1326 cm^−1^) and the phosphate (1200–900 cm^−1^) absorbance bands, respectively. The peak height ratio of absorbances at 1030cm^−1^/1020cm^−1^ was used to assess mineral crystallinity, and the relative amount of carbonate in the mineral phase was assessed by the ratio of integrated carbonate absorbance at 890–860 cm^−1^ to the phosphate absorbance (carb/min). The mineral content was normalized to total protein by the ratio of the mineral to amide I absorbance areas (min/mat). The collagen quality, related to the fibril maturation process, was calculated as the ratio of the baseline 1660/1690 cm^−1^ peak heights in the baseline amide I absorbance band. The collagen quality, or maturity, reflects the fibril maturation process and has previously been shown to be related to features in the amide I absorbance band [45].

### 3.5. Mandible Model and Post-Surgery Analyses

The animal experiment protocol was approved by the Institutional Animal Care and Use Committee (IACUC) at the University of North Carolina at Chapel Hill (IACUC ID: 18–115, 05/23/2018). To investigate craniofacial bone regeneration using H and BMP2, 5-mm critical-sized defects were generated in the mandibles of 25 Sprague Dawley male breeder rats weighing about 525 g [32,46,47]. According to Arosarena and Collins, 100 ng of BMP2 is considered a sub-optimal dose of BMP as defects exhibit minimal osteogenesis even after 8 weeks of healing [31]. This sub-optimal BMP dose was tested in our laboratory and proven to not promote any additional bone healing of the bone defect as compared to an empty or a collagen scaffold alone [33,34]. Thus, this sub-optimal dose of BMP (subBMP) was used as the negative control to evaluate the effect of H on BMP-induced bone formation with the tentative hypothesis that H would promote additional bone formation in this model. As a comparison, an optimal dose of BMP (1 µg) promotes significant bone formation in this mandible model and was used as a positive control (optBMP) [34]. Briefly, all animals were given a pre-operative dose of the antibiotic Cefazolin (10 mg/kg). Anesthesia was achieved by using Ketamine (80 mg/kg)/Xylazine (10 mg/kg). Two-cm incisions were made along the inferior border of the hemi-mandibles and the masseter muscle and the periosteum was detached to expose the ramus. Using a 5-mm-diameter trephine (Salvin Dental, Charlotte, NC, USA), a critical-sized defect was placed at the ramus about 3mm above the lower border of the mandible and 2mm distal to the incisor root [33,34]. The defects were filled with a UV cross-linked collagen sponge (Nitta Gelatin) as a scaffold. Each scaffold was precut with a 5-mm-diameter tissue punch (Miltex Inc., York, PA, USA) and was soaked uniformly in 10 µl total solution of phosphate-buffered saline and sorted into groups. Control groups included the following: (1) Collagen scaffold with subBMP (100 ng); (2) Collagen scaffold with optBMP (1 µg). Treatment groups received scaffolds loaded with (3) subBMP alone or combined with (4) H 1 µM, (5) 100 µM or (6) 500 µM or H 100 µM alone (a total of 4 treatment groups). The volume of PBS with BMP and H was a total of 10 µl. For each group, 4 rats were treated (except H 100 µM, n = 2 rats). An empty or collagen-scaffold-alone group was not implemented in this study as they have shown no difference in healing from subBMP alone [33,34]. The muscle layer was tightly sutured around the mandible with 5-0 chromic gut (Ethicon Inc., Cornelia, GA, USA) and the skin with 4-0 polypropylene suture (Ethicon), and the rats were maintained on a diet of soft rat chow and water for 4 days. Rats received buprenorphine for pain management. Euthanasia was performed 21 days post-surgery and mandibles were harvested, fixed in paraformaldehyde for 72 h and processed for microcomputed tomography (µCT) in a Scanco µCT40 scanner (SCANCO Medical AG, Bruttisellen, Switzerland) at 18 µM at 70 kV followed by demineralization with 0.5M EDTA at pH 7.4 for 8 weeks, paraffin embedding and histological processing for PSR. For PSR, 6 µm histological slides of demineralized samples were stained with Sirius red and imaged by polarized light as we previously reported [21,48]. Quantification of colors red, yellow and green was performed as described above for in vitro samples [26].

### 3.6. Statistical Analyses

For the proliferation, viability and gene expression analyses, two-tailed Student’s *t*-tests were performed for comparison of treatments with control (SAS Institute, Cary, NC, USA). For FT-IR, in vitro and in vivo PSR and in vivo BV analyses, one-way ANOVA and Tukey’s post hoc tests were used to assess significant differences among sample groups. For all statistical analyses, the significance level was set at *p* < 0.05 (GraphPad Prism, San Diego, CA, USA).

## 4. Conclusions

In conclusion, we demonstrated, for the first time, a correlation between H-induced osteogenesis in vitro and its bone regenerative capacity in vivo while emphasizing a quantifiable effect of H on collagen organization and mineralization quality as well as modulation of BMP-induced bone regeneration. This work highlights the need to further investigate the mechanism of H in BMP function and extracellular matrix biology. H may positively influence bone properties in the long term and may improve BMP’s effect clinically by promotion of its osteogenic function. Further studies in those areas are warranted, including possible modulation of BMP and its clinical inflammatory and ectopic effects.

## Data Availability

Any raw data supporting reported results can be requested by contacting the corresponding author.

## References

[1] Bauer T.W., Muschler G.F. (2000). Bone graft materials. An overview of the basic science. Clin. Orthop. Relat. Res..

[2] Bhumiratana S., Vunjak-Novakovic G. (2011). Concise Review: Personalized Human Bone Grafts for Reconstructing Head and Face. STEM CELLS Transl. Med..

[3] Kao D.W.K., Kubota A., Nevins M., Fiorellini J.P. (2012). The negative effect of combining rhBMP-2 and Bio-Oss on bone formation for maxillary sinus augmentation. Int. J. Periodontics Restor. Dent..

[4] Nevins M., Camelo M., Nevins M.L., Schenk R.K., Lynch S.E. (2003). Periodontal Regeneration in Humans Using Recombinant Human Platelet- Derived Growth Factor-BB (rhPDGF-BB) and Allogenic Bone. J. Periodontol..

[5] Garrett M.P., Kakarla U.K., Porter R.W., Sonntag V.K. (2010). Formation of Painful Seroma and Edema After the Use of Recombinant Human Bone Morphogenetic Protein-2 in Posterolateral Lumbar Spine Fusions. Neurosurgery.

[6] Selvig K.A., Sorensen R.G., Wozney J.M., Wikesjö U.M. (2002). Bone Repair Following Recombinant Human Bone Morphogenetic Protein-2 Stimulated Periodontal Regeneration. J. Periodontol..

[7] Zara J.N., Siu R.K., Zhang X., Shen J., Ngo R., Lee M., Li W., Chiang M., Chung J., Kwak J. (2011). High Doses of Bone Morphogenetic Protein 2 Induce Structurally Abnormal Bone and Inflammation In Vivo. Tissue Eng. Part A.

[8] Clarke T.C., Black L.I., Stussman B.J., Barnes P.M., Nahin R.L. (2015). Trends in the use of complementary health approaches among adults: United States, 2002–2012. Natl. Health Stat. Rep..

[9] Bagchi D., Sen C.K., Ray S.D., Das D.K., Bagchi M., Preuss H.G., Vinson J.A. (2003). Molecular mechanisms of cardioprotection by a novel grape seed proanthocyanidin extract. Mutat. Res. Mol. Mech. Mutagen..

[10] Jain M., Parmar H.S. (2010). Evaluation of antioxidative and anti-inflammatory potential of hesperidin and naringin on the rat air pouch model of inflammation. Inflamm. Res..

[11] Neto C.C. (2007). Cranberry and Its Phytochemicals: A Review of In Vitro Anticancer Studies. J. Nutr..

[12] Chen C.Y., Blumberg J.B. (2008). Phytochemical composition of nuts. Asia Pac. J. Clin. Nutr..

[13] Walter R., Miguez P.A., Arnold R.R., Pereira P.N.R., Duarte W.R., Yamauchi M. (2008). Effects of Natural Cross-Linkers on the Stability of Dentin Collagen and the Inhibition of Root Caries in vitro. Caries Res..

[14] Zhao W., Xie Q., Bedran-Russo A.K., Pan S., Ling J., Wu C.D. (2014). The preventive effect of grape seed extract on artificial enamel caries progression in a microbial biofilm-induced caries model. J. Dent..

[15] Shao Z.H., Hsu C.W., Chang W.-T., Waypa G.B., Li J., Li D., Li C.Q., Anderson T., Qin Y., Schumacker P.T. (2006). Cytotoxicity induced by grape seed proanthocyanidins: Role of nitric oxide. Cell Biol. Toxicol..

[16] Trzeciakiewicz A., Habauzit V., Mercier S., Lebecque P., Davicco M.-J., Coxam V., Demigne C., Horcajada M.-N. (2010). Hesperetin stimulates differentiation of primary rat osteoblasts involving the BMP signalling pathway. J. Nutr. Biochem..

[17] Habauzit V., Sacco S.M., Gil-Izquierdo A., Trzeciakiewicz A., Morand C., Barron D., Pinaud S., Offord E., Horcajada M.-N. (2011). Differential effects of two citrus flavanones on bone quality in senescent male rats in relation to their bioavailability and metabolism. Bone.

[18] Horcajada M.N., Habauzit V., Trzeciakiewicz A., Morand C., Gil-Izquierdo A., Mardon J., Lebecque P., Davicco M.J., Chee W.S.S., Coxam V. (2008). Hesperidin inhibits ovariectomized-induced osteopenia and shows differential effects on bone mass and strength in young and adult intact rats. J. Appl. Physiol..

[19] Habauzit V., Nielsen I.-L., Gil-Izquierdo A., Trzeciakiewicz A., Morand C., Chee W., Barron D., Lebecque P., Davicco M.-J., Williamson G. (2009). Increased bioavailability of hesperetin-7-glucoside compared with hesperidin results in more efficient prevention of bone loss in adult ovariectomised rats. Br. J. Nutr..

[20] Chiba H., Kim H., Matsumoto A., Akiyama S., Ishimi Y., Suzuki K., Uehara M. (2014). Hesperidin Prevents Androgen Deficiency-induced Bone Loss in Male Mice. Phytother. Res..

[21] Souza J.J.M., Tuin S.A., Robinson A.G., De Souza J.G.O., Bianchini M.A., Miguez P.A. (2020). Effect of Flavonoid Supplementation on Alveolar Bone Healing—A Randomized Pilot Trial. Dent. J..

[22] Shahi M., Peymani A., Sahmani M. (2017). Regulation of Bone Metabolism. Rep. Biochem. Mol. Biol..

[23] Nakashima K., Zhou X., Kunkel G., Zhang Z., Deng J.M., Behringer R.R., de Crombrugghe B. (2002). The Novel Zinc Finger-Containing Transcription Factor Osterix Is Required for Osteoblast Differentiation and Bone Formation. Cell.

[24] Xue D., Chen E., Zhang W., Gao X., Wang S., Zheng Q., Pan Z., Li H., Liu L. (2017). The role of hesperetin on osteogenesis of human mesenchymal stem cells and its function in bone regeneration. Oncotarget.

[25] Piérard G.E. (1989). Sirius Red Polarization Method is Useful to Visualize the Organization of Connective Tissues but not the Molecular Composition of their Fibrous Polymers. Matrix.

[26] Smith L.R., Barton E.R. (2014). Collagen content does not alter the passive mechanical properties of fibrotic skeletal muscle inmdxmice. Am. J. Physiol. Physiol..

[27] Hiraishi N., Maruno T., Tochio N., Sono R., Otsuki M., Takatsuka T., Tagami J., Kobayashi Y. (2017). Hesperidin interaction to collagen detected by physico-chemical techniques. Dent. Mater..

[28] Van Strijp A.J.P., Takatsuka T., Sono R., Iijima Y. (2015). Inhibition of dentine collagen degradation by hesperidin: An in situ study. Eur. J. Oral Sci..

[29] Hiraishi N., Sono R., Sofiqul I., Yiu C., Nakamura H., Otsuki M., Takatsuka T., Tagami J. (2013). In vitro evaluation of plant-derived agents to preserve dentin collagen. Dent. Mater..

[30] Islam S.M., Hiraishi N., Nassar M., Sono R., Otsuki M., Takatsura T., Yiu C., Tagami J. (2012). In vitro effect of hesperidin on root dentin collagen and de/re-mineralization. Dent. Mater. J..

[31] Arosarena O., Collins W. (2005). Comparison of BMP-2 and -4 for rat mandibular bone regeneration at various doses. Orthod. Craniofacial Res..

[32] Arosarena O.A., Collins W.L. (2005). Bone Regeneration in the Rat Mandible with Bone Morphogenetic Protein-2: A Comparison of Two Carriers. Otolaryngol. Neck Surg..

[33] Miguez P., Terajima M., Nagaoka H., Mochida Y., Yamauchi M. (2011). Role of glycosaminoglycans of biglycan in BMP-2 signaling. Biochem. Biophys. Res. Commun..

[34] Miguez P., Terajima M., Nagaoka H., Ferreira J., Braswell K., Ko C., Yamauchi M. (2014). Recombinant Biglycan Promotes Bone Morphogenetic Protein-induced Osteogenesis. J. Dent. Res..

[35] Carreira A., Lojudice F., Halcsik E., Navarro R., Sogayar M., Granjeiro J. (2014). Bone Morphogenetic Proteins. J. Dent. Res..

[36] Kuzu M., Kandemir F.M., Yıldırım S., Çağlayan C., Küçükler S. (2021). Attenuation of sodium arsenite-induced cardiotoxicity and neurotoxicity with the antioxidant, anti-inflammatory, and antiapoptotic effects of hesperidin. Environ. Sci. Pollut. Res..

[37] Kaku M., Mochida Y., Atsawasuwan P., Parisuthiman D., Yamauchi M. (2007). Post-translational modifications of collagen upon BMP-induced osteoblast differentiation. Biochem. Biophys. Res. Commun..

[38] Parisuthiman D., Mochida Y., Duarte W.R., Yamauchi M. (2005). Biglycan Modulates Osteoblast Differentiation and Matrix Mineralization. J. Bone Miner. Res..

[39] Livak K.J., Schmittgen T.D. (2001). Analysis of relative gene expression data using real-time quantitative PCR and the 2^−ΔΔCT^ Method. Methods.

[40] Mochida Y., Parisuthiman D., Pornprasertsuk-Damrongsri S., Atsawasuwan P., Sricholpech M., Boskey A.L., Yamauchi M. (2009). Decorin modulates collagen matrix assembly and mineralization. Matrix Biol..

[41] Jongwattanapisan P., Terajima M., Miguez P.A., Querido W., Nagaoka H., Sumida N., Gurysh E.G., Ainslie K.M., Pleshko N., Perera L. (2018). Identification of the effector domain of biglycan that facilitates BMP-2 osteogenic function. Sci. Rep..

[42] Pleshko N., Boskey A., Mendelsohn R. (1991). Novel infrared spectroscopic method for the determination of crystallinity of hydroxyapatite minerals. Biophys. J..

[43] Khanarian N.T., Boushell M.K., Spalazzi J.P., Pleshko N., Boskey A.L., Lu H.H. (2014). FTIR-I Compositional Mapping of the Cartilage-to-Bone Interface as a Function of Tissue Region and Age. J. Bone Miner. Res..

[44] Faillace M.E., Phipps R.J., Miller L.M. (2012). Fourier Transform Infrared Imaging as a Tool to Chemically and Spatially Characterize Matrix-Mineral Deposition in Osteoblasts. Calcif. Tissue Int..

[45] Boskey A., Camacho N.P. (2007). FT-IR imaging of native and tissue-engineered bone and cartilage. Biomaterials.

[46] Zellin G., Linde A. (1997). Importance of delivery systems for growth-stimulatory factors in combination with osteopromotive membranes. An experimental study using rhBMP-2 in rat mandibular defects. J. Biomed. Mater. Res..

[47] Arosarena O.A., Falk A., Malmgren L., Bookman L., Allen M.J., Schoonmaker J., Tatum S., Kellman R. (2003). Defect Repair in the Rat Mandible With Bone Morphogenic Proteins and Marrow Cells. Arch. Facial Plast. Surg..

[48] Yamauchi N., Nagaoka H., Yamauchi S., Teixeira F.B., Miguez P., Yamauchi M. (2011). Immunohistological Characterization of Newly Formed Tissues after Regenerative Procedure in Immature Dog Teeth. J. Endod..

